# Cosmetic and Dermatological Properties of Selected Ayurvedic Plant Extracts

**DOI:** 10.3390/molecules26030614

**Published:** 2021-01-25

**Authors:** Martyna Zagórska-Dziok, Aleksandra Ziemlewska, Tomasz Bujak, Zofia Nizioł-Łukaszewska, Zofia Hordyjewicz-Baran

**Affiliations:** 1 Department of Technology of Cosmetic and Pharmaceutical Products, Medical College, University of Information Technology and Management in Rzeszow, Kielnarowa 386a, 36-020 Tyczyn, Poland; aziemlewska@wsiz.edu.pl (A.Z.); tbujak@wsiz.edu.pl (T.B.); zniziol@wsiz.edu.pl (Z.N.-Ł.); 2 ŁUKASIEWICZ Research Network—Institute of Heavy Organic Synthesis “Blachownia”, Energetykow 9, 47-225 Kedzierzyn-Kozle, Poland; zofia.hordyjewicz@icso.lukasiewicz.gov.pl

**Keywords:** antioxidants, anti-inflammatory properties, lipoxygenase, anti-aging properties, collagenase, fibroblasts, keratinocytes, skin hydration

## Abstract

Due to the constantly growing interest in ingredients of natural origin, this study attempts to evaluate the possibility of using extracts from three Ayurvedic plants in preparations for the care and treatment of skin diseases. Therefore, studies of antioxidant properties were carried out using DPPH and ABTS radicals, obtaining 76% and 88% of these radical scavenging, respectively. A significant decrease in the intracellular level of free radicals and an increase in the activity of the antioxidant enzyme-superoxide dismutase by almost 60% were also observed. In addition, the extracts were assessed for anti-inflammatory and anti-aging properties, obtaining over 70% inhibition of lipoxygenase activity and almost 40% of collagenase. Additionally, the cytoprotective properties of the obtained extracts on skin cells, keratinocytes and fibroblasts, were demonstrated. To assess the content of biologically active compounds, HPLC-electrospray ionization (ESI)-MS/MS multiple reaction monitoring (MRM) analyses were performed. The obtained results show that all three analyzed plants are a valuable source of biologically active substances with desired properties in the context of skin cell protection. Particularly noteworthy is the extract of *Epilobium angustifolium* L., for which the most promising results were obtained.

## 1. Introduction

Natural products are an unsurpassed source of bioactive compounds and are an important economic resource for the pharmaceutical, cosmetic and food industries. Interest in plant-derived antioxidants has increased over the past few decades. Research shows that free radical species and reactive oxygen species (ROS) may cause oxidative damage. The oxidative stress contributes to the formation of inflammatory reactions in our body and plays a significant role in the etiology of chronic diseases, such as: diabetes mellitus, atherosclerosis, cancer and age-related neurological degenerative diseases [1,2]. Plant-derived polyphenols have a beneficial effect on human health due to their anti-inflammatory, anti-aging, antibacterial and antiviral properties. Hence, natural antioxidant ingredients are currently desirable compounds in the cosmetic and pharmaceutical industries due to their ability to reduce the degradation of tissues and cells in the human body by free radicals [3,4]. Our research focuses on selected plants of Asian origin, which, due to their beneficial properties, are becoming more and more popular on the Europeanmarket, both as pharmaceuticals and as innovative raw materials used in cosmetic preparations.

*Epilobium angustifolium* L. (syn. *Chamaenerion angustifolium* L.) is one of the best-known medicinal plants and has been used worldwide in traditional medicine. It is also commonly known as fireweed or rosebay willowherb. This plant is used in folk medicine for the treatment of traumatic injury, subduing inflammation, gastrointestinal and menstrual disorders, and to improve the healing of wounds, skin sores and swelling [5,6]. In vivo, aqueous extracts from *E. angustifolium* L. show a variety of beneficial properties, including analgesic, anti-inflammatory, antitumor and antiandrogenic properties. Ethanolic extracts have demonstrated bactericidal activity against both Gram-types of bacteria and cytocidal activity against fungi [7,8,9,10]. The herb is rich in polyphenolic compounds such as flavonol 3-*O*-glycosides that were isolated and identified as a strong inhibitor of COX-1, COX-2 and 5-LOX. Interestingly, this compound has strong activity (410-fold more potent than indomethacin) in the rat paw edema test, a model of acute inflammation and shows an inhibitory effect on prostaglandin biosynthesis [11,12,13]. What is more, the phytochemical screenings of *E. angustifolium* L. also revealed the presence of polyphenolic substances such as: ellagitannins, gallotannins, phenolic acids, steroids, triterpenes and fatty acids [14].

*Centella asiatica* L., commonly known as gotu kola, is a very popular plant in Ayurvedic medicine and its extracts are used to accelerate the healing of wounds, scars, psoriasis and eczema. It has also been administered for abdominal pain, indigestion and diarrhea. In Chinese medicine, gotu kola has a calming effect, improving memory and concentration, and treating depression [15,16]. The biologically active substance of the raw material is a group of triterpene compounds such as derivatives ursan (Asian and madecassic acid and their sugaresters—asiaticosideand madecassoside) and olean (terminolic acid and asiaticoside B) [17]. *C. asiatica* L. is also rich in vitamin C, vitamin B1, vitamin B2, flavonoids and carbohydrates [18,19]. In addition to herbal medicine, it is also widely used in cosmetology, where its rejuvenating and regenerating properties have been appreciated for centuries, including thanks to active substances with anti-inflammatory properties. Due to the presence of tannins and essential oils, which have a toning and stimulating effect on the skin, gotu kola is used in skin-protective preparations [20]. The flavonoids present in this plant are used in hair care products where they stimulate the peripheral circulation of the scalp and support a healthy scalp condition and prevent hair loss [21].

*Clitoria ternatea* L. commonly known as butterfly pea has been used in traditional Ayurvedic medicine as a memory enhancer, antistress, antidepressant and sedative agent [22]. Research of the in vitro and in vivo properties of different extracts of *C. ternatea* L. flowers exhibits a wide range of pharmacological aspects for the health-promoting effects. This plant possesses antioxidant, antibacterial and anti-inflammatory properties [23,24]. It is also believed to have antihyperglycemic and antihyperlipidemic [25] as well as antimicrobial activity [26]. The pro-health effect of this plant results from the presence of ternatin anthocyanins, namely A1-A3, B1-B4, C1 C4 and D1-D3, and phenolic compounds such as: quercetin and derivatives of kaempferol [27,28]. There is also more and more research on the usefulness of *C. ternatea* L. extract as a cosmetic ingredient [29,30].

As part of this paper, we investigated the potential antioxidant, anti-aging and anti-inflammatory properties of water-ethanol extracts of *Epilobium angustifolium* L., *Centella asiatica* L. and *Clitoria ternatea* L. Cytotoxicity assessments were carried out on human skin cell lines, keratinocytes and fibroblasts, using Alamar Blue, Neutral Red and Lactate Dehydrogenase assays. Due to the fact that oxidative stress plays an important role in the skin condition, the study assessed the ability of extracts to neutralize free radicals (DPPH and ABTS) and the effect of the analyzed extracts on the activity of antioxidant enzyme—superoxide dismutase. The influence of the analyzed plants on the intracellular level and activity of hydroxyl, peroxide and other reactive oxygen species was also assessed using the fluorogenic dye H_2_DCFDA. Using the HPLC-electrospray ionization (ESI)-MS/MS method, the content of various biologically active compounds in the obtained extracts was evaluated. In order to determine the anti-aging properties, the possibility of inhibiting the activity of one of the matrix metalloproteinase—collagenase, which plays an important role in the skin aging process through the degradation of collagen fibers, was assessed. In order to assess the anti-inflammatory properties of the studied extracts, the possibility of inhibiting lipoxygenase activity and the effect on protein denaturation were determined. In the final stage, the effect of the obtained extracts on skin hydration and transepidermal water loss was examined.

## 2. Results and Discussion

### 2.1. Determination of Bioactive Compounds by HPLC-ESI-MS/MS

A chromatographic method was developed to deepen the chemical structures of the active compounds. The identification of active compounds was performed by MS^2^ fragmentation of selected *m*/*z* signals. Table 1 lists active compounds detected using HPLC-ESI-MS/MS.

The obtained results of HPLC-ESI-MS/MS in the negative-ion mode revealed the presence of polyphenols, of which phenolic acids and flavonoids were the principal compounds. The observed flavonoids were quercetin and kaempferol derivatives, while phenolic acids were caffeic, quinic, gallic and caffeoylquinic acids (CQA) with two isomers: 3- and 5-CQA. Gallic acid was observed only in *Epilobium angustifolium* L. extract. Rutin was detected in *Clitoria ternatea* L. Several other flavonoid glycosides including kaempferol-3-*O*-rutinoside, kaempferol-3-*O*-glucoside, quercetin-acetyl-glucoside were also detected in the samples extracts.

In the *Epilobium angustifolium* L. extract, a dominant peak is observed for quercetin, while *Clitoria ternatea* L. extract is rich in rutin as well as quercetin and kaempferol derivatives.

**Figure 1:**
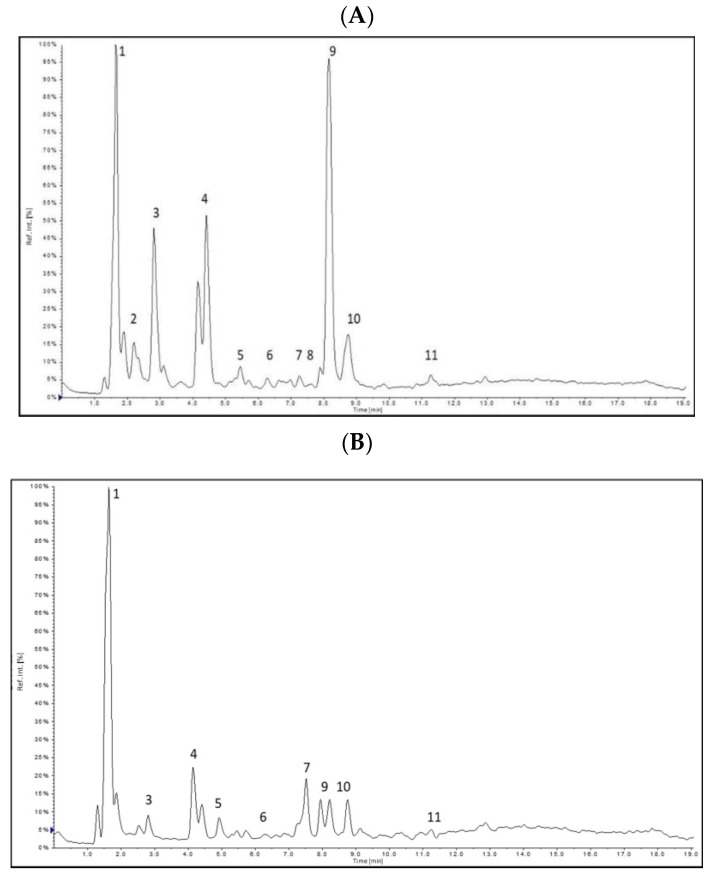

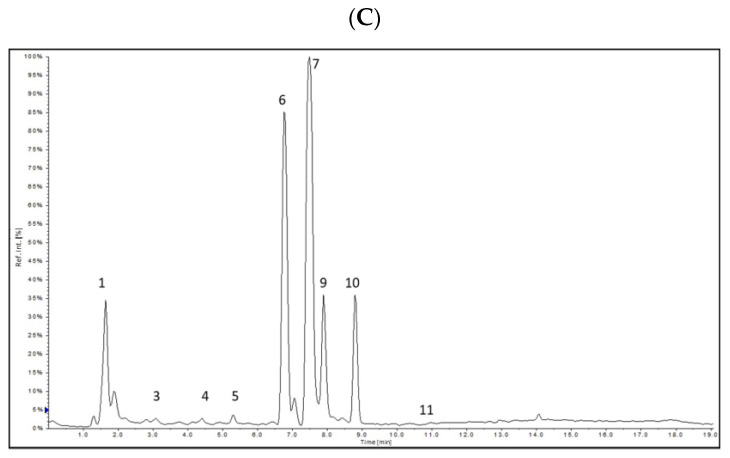
Extracted ion chromatograms (XIC) obtained for (**A**) *Epilobium angustifolium* L., (**B**) *Centella asiatica* L., (**C**) *Clitoria ternatea* L.

The extracted ion chromatogram obtained in negative-ion mode for investigated extracts are presented in Figure 1.

Quinic acid, gallic acid, caffeic acid, 3-CQA, 5-CQA and quercetin were quantified from the MRM chromatographic peak areas. As a result of the LC-ESI-MS/MS comparative analysis of the antioxidant extracts, quinic acid was found as the abundant phenolic acid representant (43.1–220.7 µg/mL), with the highest amount found in the *Epilobium angustifolium* L. *Epilobium angustifolium* L. was also reached in gallic acid (93.7µg/mL), that was below the limit of detection for other extracts. Chlorogenic acids were represented by caffeoylquinic acids with 2 isomers (3-and 5-CQA) with the highest amount of 5-CQA detected in *Epilobium angustifolium* L. extract. Quercetin was found in all three extracts with the highest content of 50 µg/mg determined for *Epilobium angustifolium* L. extract.The quantification results obtained for investigated extracts are presented in Table 2.

Total quantified active compounds contents were 467.6, 256.6 and 75.6 µg/mL for *Epilobium angustifolium* L., *Centella asiatica* L. and *Clitoria ternatea* L. extracts, respectively.

The determined phenolic content varied among the analyzed extracts. As indicated, *Epilobium angustifolium* L. extract showed the highest active compounds content. The lowest active compounds content was quantified for *Clitoria ternatea* L. however, all investigated extracts are valuable sources of biologically active substances.

### 2.2. Total Phenolic Compounds and Flavonoids

The human body is constantly exposed to adverse external factors. One of the effects of this kind of action is the excessive production of free radicals, affecting premature skin aging. Excess of free radicals can cause a lot of damage to cells and tissues. In particular, reactive oxygen species cause changes in the permeability of the cell membrane or the activity of intracellular enzymes, which can lead to serious damages or even a cell death. The most aggressive radicals, easily transported through membranes, include the hydroxyl radical, which can, among others, contribute to the breakage of the DNA chain and to the formation of modified purine bases. The appropriate line of defense is the use of antioxidants, the so-called antioxidants, thanks to which it is possible to reduce the impact of adverse free radicals on the body. One should bear in mind that many products of plant origin show strong antioxidant properties, consisting in the ability to neutralize ROS—i.e., reactive oxygen species, which include not only free radicals, but also singlet oxygen and hydrogen peroxide [5,31,32].

Plant extracts, which are one of the largest groups of cosmetic ingredients, are a rich source of biologically active substances. They can significantly affect the condition of the skin, at the same time playing a role of auxiliary substances that affect the durability or bioavailability of the preparation. They exhibit a wide range of properties which are mainly influenced by the presence of secondary metabolites contained in plants [33,34,35]. During the research carried out in the analyzed plant extracts obtained from *Epilobium angustifolium* L., *Clitoria ternatea* L. or *Centella asiatica* L., the content of biologically active compounds, including phenolic compounds and flavonoids, was assessed.

The obtained results show that the highest content of phenolic compounds (TPC) was recorded for the extract obtained from *Clitoria ternatea* L. The phenol content for this extract was 15.62 ± 0.14 mg GAE/g DW, while the lowest phenolic compounds content was found in the *Centella asiatica* L. extract, where the content of phenolic compounds was 2.96 ± 0.08 mg GAE g DW. A similar tendency was observed via the analysis of the flavonoid content (TFC) for the extract obtained from *Clitoria ternatea* L., where the recorded value was the highest and amounted to 7.26 ± 0.12 mg QE/g DW, while the lowest was for the *Centella asiatica* L. extract and amounted to 0.82 ± 0.04 mg QE/g DW. Similar values were observed for the *Centella asiatica* L. extract in the studies [36], where the levels of phenolic and flavonoid compounds were 2.86 g/100 g and 0.361 g/100 g, respectively. Intermediate values were obtained for the *Epilobium angustifolium* L. extract. Detailed data are presented in Table 3.

### 2.3. Assessment of Antioxidant Properties

#### 2.3.1. DPPH Radical Scavenging Assay

Numerous studies suggest a correlation between the content of phenolic compounds and flavonoids and the antioxidant properties of the analyzed extracts. Therefore, in the next step of the research, an analysis of the antioxidant activity of plant materials was performed using the DPPH method.

**Figure 2:**
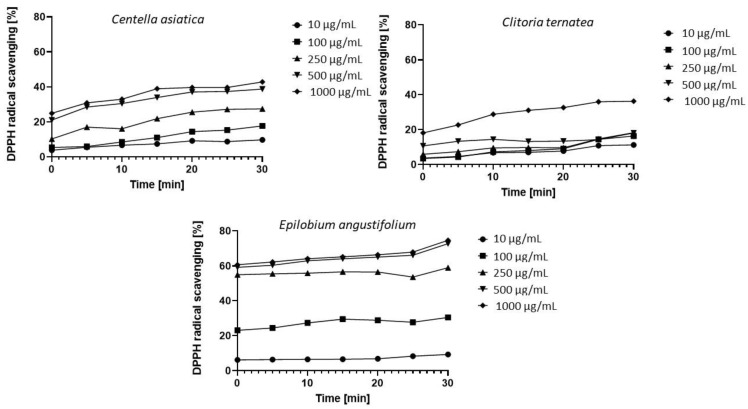
Kinetics of the absorbance changes in DPPH∙solutions in the presence of various concentrations of water-ethanol extract of *Centella asiatica* L., *Clitoria ternatea* L., *Epilobium angustifolium* L. Values are the mean of three replicate determinations (n = 3).

The tests were carried out in the concentration range from 10 μg/mL to 1000 μg/mL for a period of 30 min. Measurements were performed at five-minute intervals. As a result, seven measurement points were realized for each concentration of the tested extract. The obtained data showed that the analyzed extracts exhibit different antioxidant properties. For most of the tested concentrations, an increase in antioxidant activity was also observed over time, with the increase being the most noticeable at the highest concentrations (Figure 2).

The strongest antioxidant properties were found in the *Epilobium angustifolium* L. extract, which for the concentrations of 100 μg/mL, 250 μg/mL, 500 μg/mL and 1000 μg/mL and for all the collected measurement points, i.e., from the very beginning of the experiment to its end, showed much higher values of antioxidant activity compared to the other two extracts. Phenolic compounds are the dominant active compounds responsible for antioxidant properties of the Epilobium genus. Secondary metabolites characterized in about 25% of species of this genus indicate that the main bioactive components of this genus include flavonoids and tannins [37,38,39]. Antioxidant properties at the level of 20–40% for the concentration of 1000 μg/mL and 500 μg/mL were observed for the *Centella asiatica* L. extract. The extract owes its antioxidant properties to the flavonols—mainly quercetin and 7-glucoside of kaempferol and β-D-galacto-pyranosides of quercetin. Additionally, saponins from the extract, such as centellasaponin, asiaticoside, madecassoside, asiatic acid and madecassic acid, exhibit the ability to chelate metal ions and inhibit enzyme activity by forming complexes with metals present in the active center [40,41].

On the other hand, the weakest antioxidant properties were characteristic of the *Clitoria ternatea* L. extract. The antioxidant properties of the extract are due to, among others, such compounds as flavonoids delphinidin, kaempferol, quercetin or myricetin [42,43]. The research was carried out with the use of water-ethanol extract. In addition, analyses carried out by Kamkaen et al. 2009 showed that the water extract obtained from *Clitoria ternatea* L. has stronger antioxidant properties than the ethanol extract [44].

In the case of all tested extracts for the concentrations of 500 μg/mL and 1000 μg/mL, the observed values were the highest. The comparison of the free radical scavenging capacity of *Epilobium angustifolium* L. extract to *Centella asiatica* L. and *Clitoria ternatea* L. at these two concentrations is as follows: At the beginning of the experiment, the values for *Epilobium angustifolium* L. were 59% (for a concentration of 500 μg/mL) and 60% (for a concentration of 1000 μg/mL), which are 180% and 160% higher compared to *Centella asiatica* L., where the observed activities were respectively 21% and 23%, and by 430% and 230% higher compared to *Clitoria ternatea* L., where the values were 11% and 18%, respectively. A similar comparison after 30 min of the experiment showed that the values observed for *Epilobium angustifolium* L. were 74% (for a concentration of 500 μg/mL) and 75% (for a concentration of 1000 μg/mL), which is 90% higher and 78% compared to *Centella asiatica* L. where the observed activities were 39% and 42%, respectively, and 270% and 92% higher compared to *Clitoria ternatea* L., where the values were 20% and 39%, respectively.

This study shows that the bioactive molecules present in the analyzed extracts exhibit antioxidant properties and can be used as a prototype for the development of cosmetic and pharmaceutical raw materials.

#### 2.3.2. ABTS Radical Scavenging Assay

**Figure 3:**
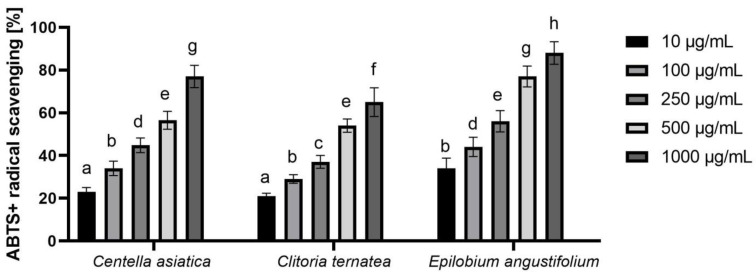
ABTS scavenging by extracts of *Centella asiatica* L., *Clitoria ternatea* L. and *Epilobium angustifolium* L. Values are the mean of three replicate determinations (n = 3). Different letters on the charts indicate significant differences between groups (*p* < 0.05).

In order to confirm the antioxidant properties of the analyzed Ayurvedic plant extracts, we also conducted another in vitro assay using the free radical ABTS. The obtained results also clearly indicate that the tested extracts have the ability to scavenge free radicals. As in the case of the first described assay, also in this test, the EAE extract showed the highest antioxidant capacity, which at the highest analyzed concentration (100 μg/mL) was able to remove 88.2% of the ABTS radical. This ability was also observed for the other two extracts, and the level of radical reduction was dose-dependent. With an increase in the concentration of the extract, and thus with an increased content of biologically active compounds, the amount of free radical ABTS decreased (Figure 3).

#### 2.3.3. Detection of Intracellular Levels of Reactive Oxygen Species (ROS)

**Figure 4:**
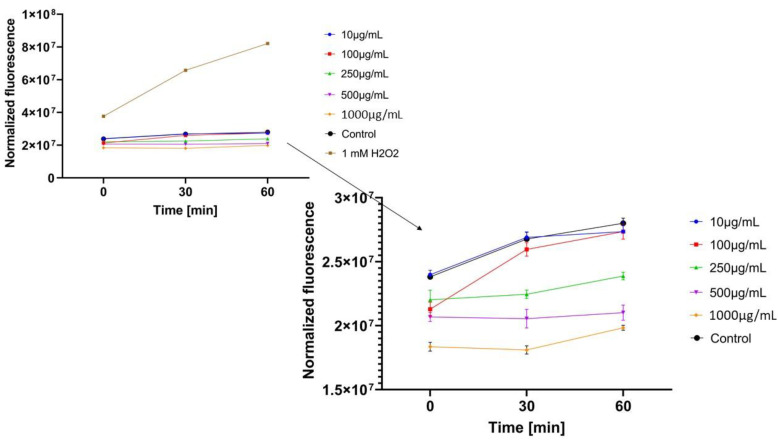
The effect of *Centella asiatica* L. extract on the 2’,7’-dichlorofluorescein (DCF) fluorescence in keratinocyte cells HaCaT. The data are expressed as the mean ± SD of 3 independent experiments, each of which consisted of 3 replicates per treatment group.

**Figure 5:**
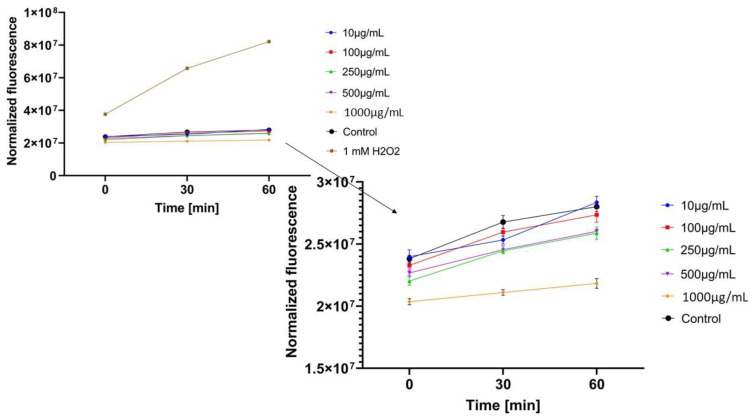
The effect of *Clitoria ternatea* L. extract on the 2’,7’-dichlorofluorescein (DCF) fluorescence in keratinocyte cells (HaCaT). The data are expressed as the mean±SD of 3 independent experiments, each of which consisted of 3 replicates per treatment group.

**Figure 6:**
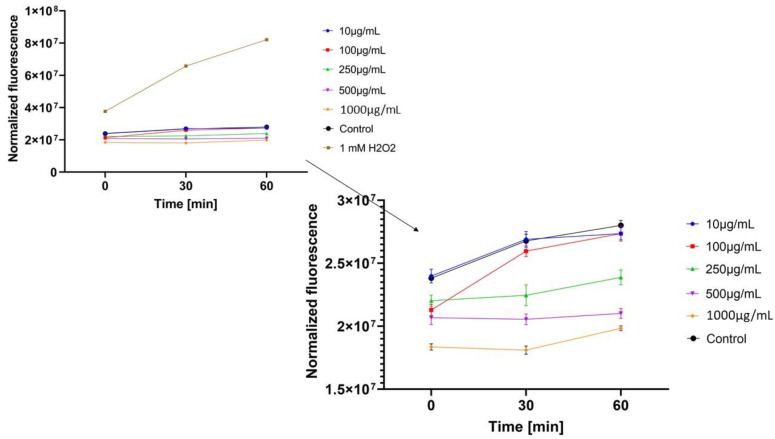
The effect of *Epilobium angustifolium* L. extract on the 2’,7’-dichlorofluorescein (DCF) fluorescence in keratinocyte cells (HaCaT). The data are expressed as the mean±SD of 3 independent experiments, each of which consisted of 3 replicates per treatment group.

**Figure 7:**
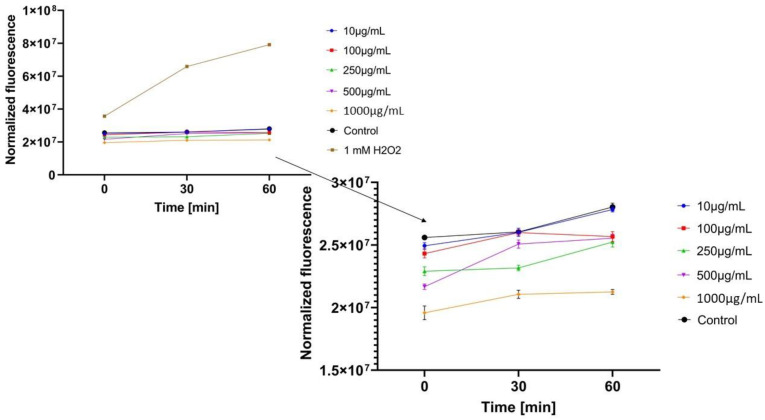
The effect of *Centella asiatica* L. extract on the 2’,7’-dichlorofluorescein (DCF) fluorescence in fibroblast cellsBJ. The data are expressed as the mean ±SD of 3 independent experiments, each of which consisted of 3 replicates per treatment group.

**Figure 8:**
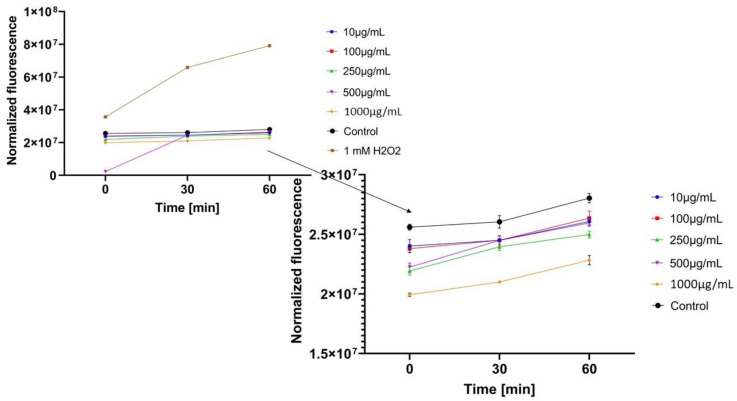
The effect of *Clitoria ternatea* L. extract on the 2’,7’-dichlorofluorescein (DCF) fluorescence in fibroblast cells(BJ). The data are expressed as the mean ± SD of 3 independent experiments, each of which consisted of 3 replicates per treatment group.

**Figure 9:**
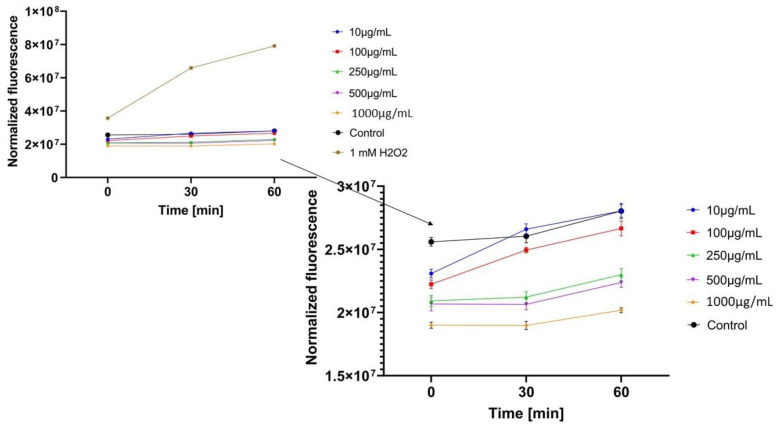
The effect of *Epilobium angustifolium* L. extract on the 2’,7’-dichlorofluorescein (DCF) fluorescence in fibroblast cells (BJ). The data are expressed as the mean ± SD of 3 independent experiments, each of which consisted of 3 replicates per treatment group.

In the next step, the influence of analyzed extracts on the generation of intracellular oxidative stress was determined. This study was performed for both keratinocytes and fibroblasts and the obtained results are shown in Figure 4, Figure 5, Figure 6, Figure 7, Figure 8 and Figure 9.

It was shown that each analyzed extract in the concentration range of 100–1000 µg/mL reduced the production of intracellular reactive oxygen species. Fluorescence observed for these concentrations of extracts was lower than the value observed for the negative control (cells without extracts). Only for the lowest analyzed extracts concentrations (10 µg/mL), intracellular oxidative stress was higher or similar with the negative control. In each of the analyzed samples, it was also shown that an increase in the extract’s concentration reduces the production of intracellular ROS.

ROS production in keratinocyte cells was most effectively inhibited by *Centella asiatica* L. (GCE) and *Epilobium angustifolium* L. (EAE) extracts. High, but lower efficiency in reducing oxidative stress at the cellular level was observed for *Clitoria ternatea* L. extract (KTE). In the case of GCE and EAE extracts at the concentrations of 250–1000 µg/mL, a more than two-fold reduction of ROS production was observed at the beginning of the experiment and nearly four-fold after 60 min incubation, in relation to 1mM H_2_O_2_ solution (positive control).

Regarding the study with fibroblast cells, the most favorable results were obtained for *Epilobium angustifolium* L. extract. *Centella asiatica* L. and *Clitoria ternatea* L. extracts showed significantly lower ability to inhibit ROS production. For the EAE sample, value of the analyzed parameter was reduced more than two-fold compared to the positive control after the beginning of the incubation and more than four-fold after 60 min incubation.

Free radicals are one of the factors that plays a major role in the skin aging process and can also be the cause of many dermatological diseases [45]. Preventing the effects of oxidative stress caused by free radicals is possible by applying antioxidants on the skin or taking them internally. Plants are a rich source of these substances, from which antioxidants are isolated through the extraction process [45,46]. In the studies of Moolsap et al. [47], it has been shown that *Centella asiatica* L. extracts effectively reduce the production of ROS in keratinocyte cells. The antioxidant potential of this extract in vitro and in vivo models has also been demonstrated in numerous studies [36,48,49,50]. The effectiveness of the water-ethanol *Clitoria ternatea* L. extract as an inhibitor of free radicals generated by UV radiation and H_2_O_2_ in keratinocytes was demonstrated by Zakkaria et al. [51], indicating the significant role of anthocyanins and flavanols in this process as the main components of the extract. Analyzing the literature, no reports on the antioxidant potential of *Epilobium angustifolium* L. were found. Results obtained for *Epilobium angustifolium* L., better than for *Clitoria ternatea* L. and *Centella asiatica* L., which are indicated as plants with a high antioxidant potential, may prove the high potential of *Epilobium angustifolium* L. to minimize oxidative stress in cells. This is a premise for further research on the use of this plant in cosmetics and medicine.

#### 2.3.4. Determination of Superoxide Dismutase (SOD) Activity

**Figure 10:**
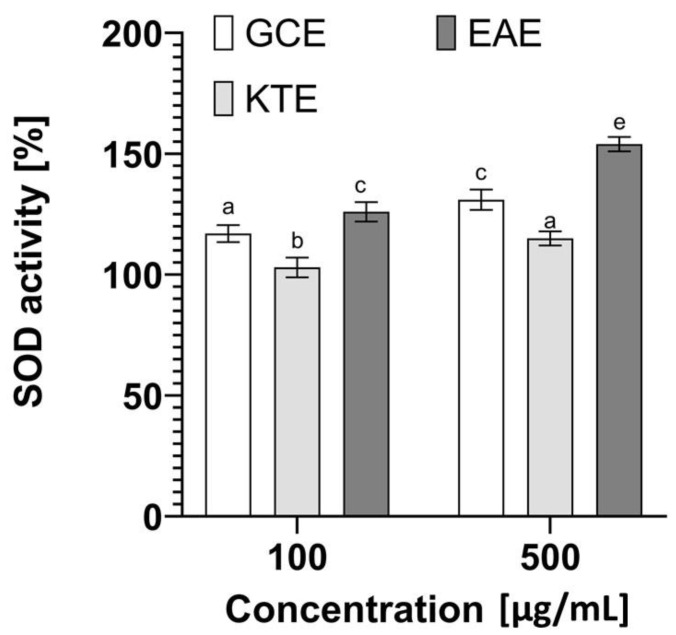
Effect of *Centella asiatica* L. (GCE), *Clitoria ternatea* L. (KTE) and *Epilobium angustifolium* L. (*EAE*) extracts on superoxide dismutase activity. Data are the mean ± SD of three independent experiments, in which each concentration was tested in duplicate. Different letters on the charts indicate significant differences between groups (*p* < 0.05).

In order to confirm the antioxidant activity of the obtained extracts, the activity of the antioxidant enzyme—superoxide dismutase (SOD) was measured. This metalloprotein plays an important role in the first stage of antioxidant defense and protects cells against various types of damage caused by free radicals [52]. The analyses carried out as part of this paper showed that the analyzed extracts are characterized by a different ability to influence the activity of this enzyme (Figure 10). Particularly promising results were obtained in the case of *Epilobium angustifolium* L., which at a concentration of 500 µg/mL shows the ability to increase the activity of this enzyme by almost 60%, which can significantly affect the fight against free radicals. Approximately 30% increase in superoxide dismutase activity was also obtained in the case of the extract of *Centella asiatica* L., while *Clitoria ternatea* L. had only a small effect on the increase in the activity of the SOD, which may indicate that its antioxidant properties shown in the analyses above are related to a different mechanism. The obtained results correlate with other analyses carried out as part of this study assessing the antioxidant capacity (with the use of DPPH and H_2_DCFDA), indicating that the tested plants, especially *Epilobium angustifolium* L., can significantly contribute to the protection of cells against oxidative stress, lipid peroxidation or damage. The ability of biologically active compounds in plant extracts to influence the number of free radicals is extremely important in the context of skin cells. These radicals can contribute to changes in the skin structure and significantly affect its aging processes [53].

### 2.4. Cell Viability Assays

In our research, the cytotoxicity of analyzed extracts against fibroblasts and keratinocytes—skin building cells—was evaluated in vitro using three types of assay (Neutral Red uptake assay, Alamar Blue test and LDH cytotoxicity assay). Neutral Red uptake and Alamar Blue tests, in which the assessment of the cytotoxicity of the tested substances based on the analysis of its influence on the integrity of lysosomal membranes and the activity of cell mitochondria, are the most popular cytotoxicity tests used in many biomedical and industrial applications.

**Figure 11:**
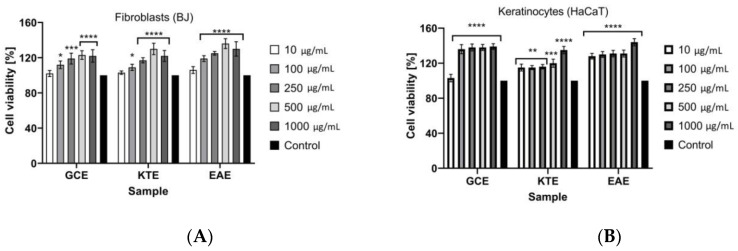
The effect of *Centella asiatica* L. (GCE), *Clitoria ternatea* L. (KTE) and *Epilobium angustifolium* L. (EAE) (1–1000 μg/mL) on Neutral Red Dye uptake in cultured (**A**) fibroblasts (BJ) and (**B**) keratinocytes (HaCaT) after 24 h of exposure. Data are the mean ± SD of three independent experiments, each of which consists of four replicates per treatment group. For BJ **** *p* < 0.0001, *** *p* = 0.0002, * *p* < 0.0112 versus the control (100%). For HaCaT **** *p* < 0.0001, *** *p* = 0.0006, ** *p*< 0.0084 versus the control (100%).

In the Neutral Red uptake test (Figure 11A,B), it was observed that analyzed extracts did not show any cytotoxic activity against both keratinocytes and fibroblasts. It was noticed that an increase in the concentration of extracts increased the proliferation of cells. At the lowest tested concentration (10 µg/mL) of each extract, the viability of fibroblasts was about 105%. The highest viability of fibroblasts was observed at the extract’s concentration of 500 and 1000 µg/mL. For GCE extract at these concentrations, cell proliferation reached about 120%. For KTE and EAE extracts at concentrations of 500 µg/mL the results were 130% and 135%, respectively. At the KTE and EAE extracts concentration of 1000 µg/mL a slight decrease in fibroblasts proliferation was observed to about 120% and 130%, respectively. The strong increase of proliferation was also observed for keratinocytes. For GCE extract at the concentration of 100–1000 µg/mL the viability of these cells was about 140%. For KTE extract, in the concentration range of 10–500 µg/mL, value of the analyzed parameter reached about 120% and at the concentration of 1000 µg/mL strong increase in keratinocyte proliferation to about 135% was observed. The highest cell proliferation of keratinocytes was observed for EAE extract at the concentration of 1000 μg/mL and this increase reached 145%. At the lower concentrations of this extract, the viability of keratinocytes was high and reached about 130%.

**Figure 12:**
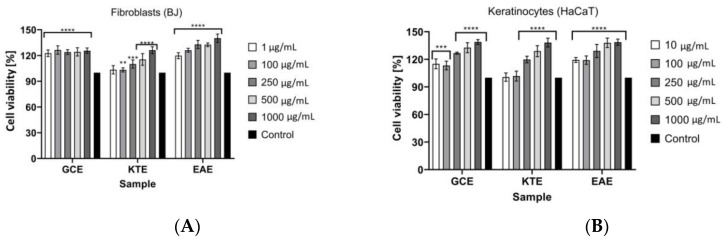
The reduction of resazurin after 24 h exposure to the *Centella asiatica* L. (GCE), *Clitoria ternatea* L. (KTE) and *Epilobium angustifolium* L. (EAE) extracts in cultured (**A**) fibroblasts (BJ) and (**B**) keratinocytes (HaCaT). Data are the mean ± SD of three independent experiments, each of which consists of three replicates per treatment group. For BJ **** *p* < 0.0001, *** *p* = 0.0001, ** *p* < 0.007 versus the control (100%). For HaCaT **** *p* < 0.0001, *** *p* = 0.0005 versus the control (100%).

In the Alamar Blue test (Figure 12A,B) it was also indicated that analyzed extracts did not show any cytotoxic effects both for keratinocytes and fibroblasts. The strongest cell proliferation of fibroblast was noted for EAE extract. At the concentration of 1000 µg/mL the cell viability reached about 140%. In the concentration range of 100–500 μg/mL the value of the analyzed parameter was about 125–130%. For GCE extract it was not observed statistically significant differences and in each analyzed concentration cell viability reached about 125%. The lowest ability to increase fibroblast proliferation was observed for KTE extract. At the lowest tested concentrations of this extract, cell viability at the level of about 105–110% was observed, while at the concentration of 1000 µg/mL, an increase to about 125% was noted. Analogical results were observed for keratinocytes. The highest cell viability was observed at the highest concentrations of analyzed extracts, but no significant differences were noted between them. In the concentration range of 500–1000 µg/mL the increase in cell proliferation reached about 135–140% for GCE, KTE and EAE samples. At the lowest analyzed concentrations, the weakest proliferation properties were observed for KTE extract.

**Figure 13:**
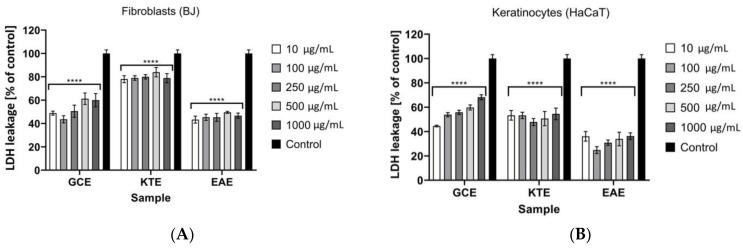
The release of LDH after 24h exposure to *Centella asiatica* L. (*GCE*), *Clitoria ternatea* L. (*KTE*) and *Epilobium angustifolium* L. (EAE) extracts in cultured (**A**) fibroblasts (BJ) and (**B**) keratinocytes (HaCaT). Data are the mean ± SD of three independent experiments, each of which consists of three replicates per treatment group. **** *p* < 0.0001 versus the control (100%).

Cytotoxic properties of analyzed extracts using LDH test were also investigated in our research (Figure 13A,B). As a result of the cell membrane damage and/or cell death, lactate dehydrogenase is released from the cells into the environment but under physiological conditions release of LDH is not observed. In this method, the increase in activity of released LDH is a measure of the cytotoxic activity of the analyzed substance. At each analyzed extract concentrations cytotoxic effects and significant membranes damage were not observed. For GCE extract, an increase in LDH release with an increase of extract concentration was observed both for keratinocytes and fibroblasts. For KTE and EAE extracts, values of the analyzed parameter as a function of extracts concentration were not significantly different. The strongest ability to decrease LDH release was observed forEAE extract. In relation to the control sample, a decrease in LDH release by about 60% was noted, both for keratinocytes and fibroblasts. GCE extract showed the lowest ability to inhibit LDH release. A reduction of about 20% in relation to the control for fibroblasts and of about 40% for keratinocytes was observed.

Plant extracts are a valuable source of many bioactive substances that are responsible for their ability to increase the proliferation of keratinocytes and fibroblasts [54]. As Csepregi et al. [55] have shown, many plants have proven properties to increase the viability and migration of skin cells. This action is one of the most important factors in the wound healing and prevention of skin aging processes. Organic acids, polyphenols, flavonoids, vitamins and proteins are the main active ingredients of plants that are responsible for increase of proliferation of skin cells [55,56]. Among many active substances of plants, the strongest ability to improve the skin cells proliferation is shown by, e.g., caffeic acid and its derivatives, coumaric acid, gallic acid, rutin, quercetin and their derivatives [55,56]. As literature data shows [57,58,59,60], one of the most valuable plant extracts with proliferative properties of various kinds of cells is *Centella asiatica* L. extract (Gotu kola). Aqueous, ethanolic and methanolicGotu kola extracts not only increase the skin cells viability but also significantly affect their migration properties, which gives this plant strong regenerative properties that are used in wound-healing processes. Additionally, the strong ability to stimulate fibroblasts proliferation leads to increase of collagen and elastin production in the skin and Gotu kola is considered in cosmetics as the strong and effective anti-aging ingredients. Results of research about proliferative properties of *Clitoria ternatea* L. and *Epilobium angustifolium* L. was not found in the literature. Results of our research indicate similar or better properties of these plants to increase the viability of fibroblasts and keratinocytes compared to Gotu kola. Therefore, it can be concluded that these plants will be characterized by an equally strong regenerative effect on the skin, as shown in numerous studies for Gotu kola. The observed most favorable properties of *Epilobium angustifolium* L. extract may be due to the presence of gallic acid and caffeic acid derivatives in this extract, which were not identified in the other tested extracts.

### 2.5. Determination of Anti-Collagenase Activity

**Figure 14:**
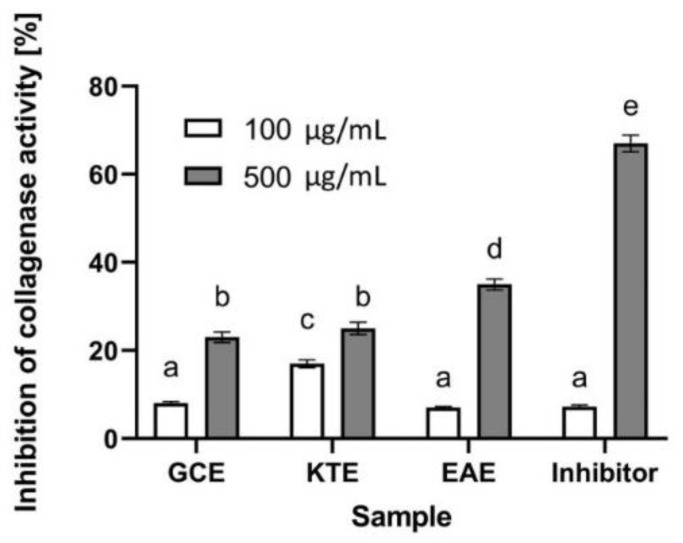
Influence of *Centella asiatica* L. (GCE), *Clitoria ternatea* L. (KTE) and *Epilobium angustifolium* L. (EAE) extracts on collagenase inhibition. Data are the mean ± SD of three independent experiments, each of which consists of three replicates per treatment group. Different letters on the charts indicate significant differences between groups (*p* < 0.05).

Collagen is a protein that ensures adequate strength, elasticity and hydration of the skin. It plays an important role not only in the aging process of the skin, but also in wound healing and skin regeneration [61]. The reduced content of this protein in the skin may contribute to the formation of wrinkles by weakening the bond between the dermis and the epidermis, which significantly affects the appearance of the skin [62]. An enzyme that contributes to the degradation of the collagen structure in the skin is collagenase. Its increased activity may be the result of free radicals and UV radiation, as well as internal and genetic conditions [61]. Therefore, in order to assess the potential use of the studied extracts in the fight against skin aging, the possibility of extracts from *Centella asiatica* L., *Clitoria ternatea* L. and *Epilobium angustifolium* L. for inhibiting the activity of the collagenase enzyme was investigated. Analyses of collagenase inhibition capacity by extracts from three plants carried out as part of this study showed that all tested plants have the ability to inhibit this enzyme, which indicates their potential anti-aging properties. It has been observed that the inhibitory abilities of this enzyme depend both on the plant used and the concentration of the extract. The greatest inhibitory properties have *Epilobium angustifolium* L., which is capable of almost 40% inhibition of the activity of this enzyme at a concentration of 500 µg/mL. The other two plants (in the higher tested concentration) showed a lower ability to inhibit this matrix metalloproteinase (slightly over 20%) (Figure 14). The fact that secondary metabolites and plant extracts can inhibit collagenase degradation has already been proven by many authors. It is the result of a wide range of various biologically active compounds, such as flavonoids, tannins, phenolic acids and tocopherols [63]. The presence of carbonyl and hydroxyl groups in the molecules of plant flavonoids enables them to form complexes with metal ions, thanks to which they can bind with metalloenzymes such as collagenase [64]. This property contributes to the possibility of altering or inhibiting various metabolic pathways [63]. Chromatographic analyses of the extracts obtained by us show the presence of various polyphenolic compounds such as quercetin and kaempferol derivatives and phenolic acids such as caffeic, quinic, gallic and chlorogenic (5-caffeoylquinic acid). Literature studies show that these compounds can prevent collagen degradation by inhibiting collagenase activity. This effect can be observed both during collagen degradation because of ongoing inflammation, as well as in skin that undergoes photoaging [65]. The presence of quercetin in the extract of *Epilobium angustifolium* L. may significantly affect the anti-aging properties of this plant, as research shows that it is one of the most effective flavonoids with anti-collagenase activity [65]. Alsorutin and caffeic acid present in the obtained extracts are known to counteract the effects of aging by inhibiting the activity of this enzyme [66].

### 2.6. Assessment of Anti-Inflammatory Potential

**Figure 15:**
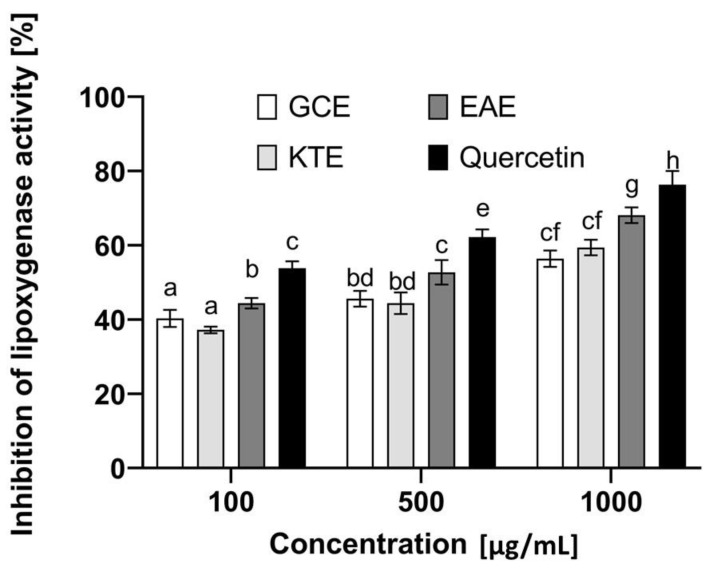
Influence of *Centella asiatica* L. (GCE), *Clitoria ternatea* L. (KTE) and *Epilobium angustifolium* L. (EAE) extracts on lipoxygenase inhibition. Data are the mean ± SD of three independent experiments, each of which consists of two replicates per treatment group. Different letters on the charts indicate significant differences between groups (*p* < 0.05).

**Figure 16:**
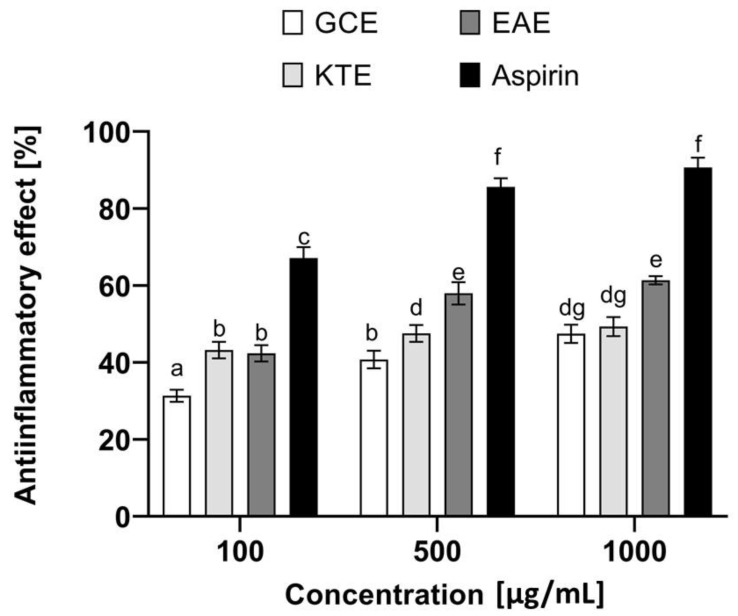
Anti-inflammatory effect of *Centella asiatica* L. (GCE), *Clitoria ternatea* L. (KTE) and *Epilobium angustifolium* L. (EAE) extracts. Data are the mean ± SD of three independent experiments, each of which consists of three replicates per treatment group. Different letters on the charts indicate significant differences between groups (*p* < 0.05).

An extremely important aspect in the context of the healthy appearance of the skin and its proper functioning is the ongoing inflammation causing numerous skin disorders. Lipoxygenases are important enzymes that play a significant role in various types of inflammation. These enzymes catalyze the oxidation reactions of polyunsaturated fatty acids such as arachidonic and linoleic acids. On the other hand, lipid oxidation is responsible for the initiation of subsequent biological reactions and activation of various cell signaling mechanisms [67]. Literature data indicate that phenolic compounds may contribute to the inhibition of the activity of these enzymes, which indicates the possibility of their use in the fight against inflammatory skin diseases [68]. Therefore, as part of the research, analyses were carried out to assess the potential anti-inflammatory properties of extracts obtained from *Centella asiatica* L., *Clitoria ternatea* L. and *Epilobium angustifolium* L. (Figure 15). For this purpose, the effect of the analyzed extracts on the inhibition of lipoxygenase activity and the impact on proteins denaturation was investigated. These are two commonly used in vitro methods to evaluate the anti-inflammatory properties of various herbal extracts [69]. The analyses carried out as part of this paper show that the plants tested exhibit various anti-inflammatory properties, and the effect is closely related to the concentration used. As shown in Figure 15, the most effective of the concentrations tested is 500 µg/mL. The plant extract that shows the greatest ability to inhibit the activity of lipoxygenase is the water-ethanol extract of *Epilobium angustifolium* L., which inhibits the activity of this enzyme to a comparable degree as the commonly known lipoxygenase inhibitor—quercetin (70.5% and 79%, respectively), which was used in our research as a control compound. As shown by other authors, the effect of this compound is related to the lengthening of the delay period, a rapid decrease in the initial rate after the lag phase has been overcome and the time-dependent inactivation of the enzyme [69]. The presence of various biologically active compounds confirmed in our study certainly contributes to the ability to inhibit this enzyme, because, as reported in the literature, many plant compounds show the ability to inhibit various isoforms of lipoxygenases [70,71]. However, it should be noted here that the presence of many isoforms of this enzyme contributes to the fact that the development of a selective inhibitor is extremely difficult. Nevertheless, the search for plants that can, at least to some extent, influence the activity of this enzyme and contribute to reducing inflammation is highly desirable. To evaluate the anti-inflammatory properties of the extracts from three tested plants, their ability to inhibit protein denaturation was also tested. This denaturation causes changes in the spatial structures of proteins and the loss of their biological properties, which also correlates with the formation of various types of inflammatory disorders. As shown in Figure 16, the analyzed extracts can inhibit the protein denaturation process in a dose-dependent manner. Similarly, to the previously described test (inhibition of lipoxygenase), the most promising results were obtained for the extract of *Epilobium angustifolium* L., which at a concentration of 1000 µg/mL was able to inhibit denaturation by 61.5%. *Centella asiatica* L. and *Clitoria ternatea* L. extracts were characterized by slightly lower effectiveness, which inhibited BSA denaturation by 49% and 51%, respectively. The results obtained as part of these analyses indicate that the analyzed extracts can be perceived as a valuable source of compounds that can support the fight against minimizing the effects of inflammatory diseases.

### 2.7. Transepidermal Water Loss (TEWL) and Skin Hydration Measurements

**Figure 17:**
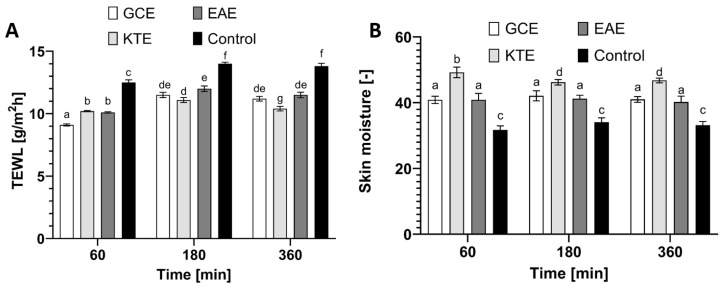
Influence of *Centella asiatica* L. (GKE), *Clitoria ternatea* L. (KTE) and *Epilobium angustifolium* L. (EAE) extracts (10 µg/mL) on transepidermal water loss (TEWL) (**A**) and skin hydration (**B**). The determinations were made in five replicates. Different letters on the charts indicate significant differences between groups (*p <* 0.05).

The functional barrier to the rate of water loss by the body is intact skin, more specifically the stratum corneum. This skin layer prevents the body from losing important components such as ions, water and serum proteins, but is not completely impermeable to compounds applied directly to the skin surface. The permeability barrier is influenced by various external and internal factors such as physical stressors, climatic conditions and microorganisms [72,73]. Currently, in vivo methods are used to monitor the physical properties of the skin barrier, including the quantification of epidermal water loss (TEWL). In the healthy stratum corneum, the water content is usually 10–20% [74,75]. Currently, raw materials are sought that will positively affect the properties of the skin. Our study determined the effect of *Centella asiatica* L., *Clitoria ternatea* L. and *Epilobium angustifolium* L. water-ethanol extracts on basic skin parameters such as skin hydration and TEWL. The results are shown in Figure 17.

The application of plant extracts to the skin improves both skin hydration and TEWL levels. These measurements were performed at various time intervals: after 60, 180 and 360 min. The conducted research showed that after the application of the analyzed substances to the skin, a significant decrease in TEWL was noted in relation to the control field. After 60 min of the application of the extracts to the skin, the greatest decrease in TEWL was recorded for the *Centella asiatica* L. extract by about 30% compared to the control field. Moreover, after 360 min of use, the tendency of TEWL decrease was still high (approximately 25% compared to the control for the *Clitoria ternatea* L. extract). This means that the TEWL lowering effect lasts long after the analyzed samples are applied to the skin of the forearm. It was also noticed that the application of the tested plant extracts affects the level of skin hydration. The most favorable properties were observed for the *Clitoria ternatea* L. extract. After 60 min of substance application, the level of hydration increased by 70% compared to the control field. With the passage of time, the trend in the level of hydration remained at the same level for all tested extracts.

Plant extracts contain a wide range of beneficial substances belonging to secondary metabolites, as well as proteins, carbohydrates and vitamins. In their molecules, they have hydroxyl groups that can form a hydrogen bond with the water associated with the water in the epidermis. Proteins and carbohydrates due to their high molecular weight may act as an occlusive film on the skin surface causing a decrease of transepidermal water loss [76]. The tested plants contain compounds responsible for increasing the hydration of the skin and preventing transepidermal leakage of water from the epidermis, thus having a beneficial effect on the human body [14,17,19,27,28]. They can be successfully used as ingredients in cosmetics.

## 3. Materials and Methods

### 3.1. Plant Material and Extraction Procedure

Plant material was purchased from a local certified herbal store. Herbs were collected on controlled and ecological plantations. No chemical fertilizers nor plant protection products were used for the cultivation. In addition, after obtaining the plant material, a preliminary selection was carried out, paying particular attention to chemotaxonomic factors. All plants were collected in 2019. The flowers of KTE were collected in June, while the herb of the EAE and GCEin July.

As part of this work, extracts of *Centella asiatica* L., *Clitoria ternatea* L. and *Epilobium angustifolium* L. were prepared. KTEflowers and GCE and EAE herbs were used to prepare the extracts. For this purpose, ultrasound assisted extraction (UAE) was used. A mixture of water and ethanol (80:20 *v*/*v*) was used as the extractant. A total of 5 g of plant material and 95 g of mixture were used to prepare each of the extracts. The ultrasonic cleaner mixtures were extracted at room temperature (about 22 °C) for 80 min (8 cycles of 10 min). The resulting extracts were then collected and filtered three times through Whatman No. 10 filter paper using vacuum filtration. The extracts were then evaporated using a vacuum evaporator and stored in the dark at 4 °C until further analysis.

### 3.2. Determination of Biologically Active Compounds

#### 3.2.1. Determination of Bioactive Compounds by HPLC-ESI-MS/MS

The obtained extracts were analyzed to determine their main bioactive compounds using a HPLC (DionexUltiMate 3000 RS, Thermo Fisher Scientific, Sunnyvale, CA, USA), coupled to mass spectrometer (4000 QTRAP, AB Sciex, Concord, ON, Canada), equipped with an electrospray ionization source (ESI) and a triple quadrupole-ion trap mass analyzer, working in the multiple reaction monitoring (MRM) scan mode Chromatographic separation was achieved with a gradient reverse-phase system. The 100 × 4.6 mm chromatographic column Kinetex 3.5 µm XB-C18 100 Å with iso-butyl side chains and with TMS endcapping stationary phase used with similar composition guard column, was purchased from Phenomenex, and maintained at 30°C. A binary solvent system comprising 0.1% (*v*/*v*) aqueous formic acid as solvent A and methanol as solvent B was used under gradient mode during 19.1 min of the run time. The elution conditions applied were as follow: 0.0–15.0 min 25–100% B, 15.0–17.0 min 100% B, 17.0–17.1 min 100–25% B, 17.1–19.1 min 25% B. The flow rate of the mobile phase was 0.6 mL/min and injection volume 10 μL.The eluent was monitored by electrospray ion mass spectrometer (ESI-MS) under negative ion mode and scanned from *m*/*z* 20 to 1000 Da. For quantification analysis the triple quadrupole MS detector was working in multiple reaction monitoring (MRM) scan mode. Optimal mass analyzer conditions and the selection of product ions for individual compounds were determined experimentally.For this purpose, standard solutions of investigated compounds (1 ng/mL) in mobile phase composition were introduced using infusion pump operating in constant sample delivery. After ensuring that the correct precursor ion was selected, declustering potential (DP), entrance potential (EP), collision cell exit potential (CXP), collision energy (CE) was optimized for each MRM transition (Table A1). Two MRM transitions were monitored: one for quantification and one for confirmation. The MS parameters were set as follows: capillary temperature of 600°C, curtain gas at 35 psi, nebulizer gas at 60 psi and drying gas at 50 psi. Negative ionization mode source voltage −4500 V was applied for determination of phenolic compounds. Nitrogen was used as curtain and collision gas. Data analysis was processed with Analyst 1.5.1 software. The identification of selected compounds was done by molecular mass and fragment of anion entries of each individual compound and confirmed by MS^2^ fragmentation. The identities of 11 compounds were determined along with their chemical formula, deprotonated molecular ions and the characteristic fragment ions for each individual peaks. 6 compounds were quantified based on the calibration curve generated using peak areas of the most intense MRM transitions of analytical standards. The linearity of the detector response for quantified compounds was demonstrated by injection of calibration standards at eight concentration levels ranging from 0.1 μg/mL to 20 μg/mL.Calibration curves were linear with the coefficients of correlation (R) greater than 0.99. In case the samples did not fall in the linear range of the MS detector, the samples were diluted.

Analytical standards of quinic acid, gallic acid, caffeic acid, caffeoylquinic acids (CQA, two isomers: 3- and 5-CQA) and quercetin were purchased from Sigma-Aldrich (Saint Louis, USA). All standards used were of analytical grade (≥99% purity).

Standard stock solutions were prepared by accurately weighing and dissolving 200 mg of each standard in 10 mL LC-MS grade methanol to give a concentration of 20 mg/mL (20,000 ppm). Serial dilutions of 20 ppm, 15 ppm, 10 ppm, 5 ppm, 1 ppm, 0.5 ppm, 0.2 ppm and 0.1 ppm were then made using LC-MS grade methanol solution.

LC-MS/MS assay was performed in triplicate. Obtained data were presented as means ± standard deviations. 

#### 3.2.2. The Determination of the Total Phenolic Content (TPC)

The assessment of total phenolic compounds content in extracts were analyzed spectrophotometrically using the Folin–Ciocalteu method with some modification [77]. The procedure described by Zagórska-Dziok et al. [78] was used. This method depends on the reduction of Folin’s reagent by phenols to a mixture of blue oxides which have a maximal absorption in the region of 740 nm. The 300 μL tested extract sample and a standard solution of varying concentrations were mixed with 1500 μL of 10% ethanolic solution of Folin–Ciocalteu reagent. The deionized water was used for dilution and as a control sample. After incubation for 6 min in the dark at room temperature, 1200 μL of 7.5% sodium carbonate solution was added to each sample followed by mixing and incubation for 2 h. The absorbance was measured at λ = 740 nm using a spectrophotometer AquamateHelion (Thermo Scientific, Waltham, MA, USA). The total phenolic compounds concentration was calculated from a gallic acid (GA) calibration curve (10–100 mg/mL). Data were expressed as gallic acid equivalents (GA)/g of extract averaged from three independent measurements.

#### 3.2.3. The Determination of the Total Flavonoids Content (TFC)

The assessment of total flavonoid content in the analyzed extracts was performed spectrophotometrically using aluminum nitrate nonahydrate by method adopted by Matejić et al. with modifications [79]. According to this method and procedure described by Nizioł-Łukaszewska et al. [80], a reaction mixture was prepared containing 80% C_2_H_5_OH, 10% Al(NO_3_)_3_ × 9 H_2_O and 1M C_2_H_3_KO_2_. Then, 2400 μL of the previously prepared reaction mixture was mixed with 600 μL of tested extract sample in various concentrations. The deionized water was used for dilution and a control sample. After incubation for 40 min at room temperature, the absorbance was measured at λ = 415 nm with UV/VIS spectrophotometer AquamateHelion (Thermo Scientific, Waltham, MA, USA). Quercetin was used as a standard for calibration curve and the results were expressed as Quercetin equivalents (Qu/g) of extract averaged from three independent measurements.

### 3.3. Assessment of Antioxidant Properties

#### 3.3.1. DPPH Radical Scavenging Assay

The 1,1-diphenyl-2-picryl hydrazyl radical (DPPH) was used for determination of free radical-scavenging activity of the analyzed plant extracts. This method was described by Brand-Williams et al. [79]. Procedure described by Nizioł-Łukaszewska et al. was used [81]. Then, 33 μL of tested plant samples at the concentrations range of 10 μg/mL to 1000 μg/mL were added to 167 μL methanol solution of DPPH (4 mM) in a 96-well plate. The mixture was shaken vigorously, and absorbance was measured at λ = 516 nm with FilterMax F5 microplate reader(Thermo Fisher Scientific, Waltham, MA, USA). Measurements were carried out in triplicate for each sample. As a control, distilled water mixed with DPPH solution was used. The scavenging activity on the DPPH radical was expressed as inhibition percentage using the following equation:

$$\%\;DPPH•scavenging=\frac{Abs\;control-Abs\;sample}{Abs\;control}\times \;100$$

where *Abs* control is the absorbance of the control sample (containing all reagents except the test extract or standard), and *Abs* sample is the absorbance of the test extract or standard.

#### 3.3.2. ABTS+ Scavenging Assay

Scavenging of ABTS•+ (2,2′-Azino-bis(3-ethylbenzothiazoline-6-sulfonic acid) diammonium salt) free radical was evaluated according to procedure described by Gaweł-Bęben et al. [82]. Then, 19.5 mg ABTS and 3.3 mg potassium persulfate was mixed with 7 mL phosphate buffer (pH = 7.4) and dissolved for 16 h in darkness. The solution was diluted to the absorbance at the level of about 1.0 (measurement at λ = 414 nm). Then, 20 µL of GCE, KTE and EAE was mixed with 980 µL of diluted ABTS•+ solution and then incubated for 10 min in darkness. The decrease in ABTS•+ absorbance was measured at λ = 734 nm using a UV/VIS spectrophotometer AquamateHelion (Thermo Fisher Scientific, Waltham, MA, USA). Distilled water was used as a blank. The ABTS•+ scavenging was calculated from the equation:

$$\%\;\mathrm{of}\;\mathrm{ABTS}•+\;\mathrm{scavenging}\;=1-\;\frac{As}{Ac}\;\times \;100\;\%$$

where:

*As*—absorbance of the sample;
*Ac*—absorbance of the control sample.
Measurements were carried out in triplicate for each extract sample.

#### 3.3.3. Detection of Intracellular Levels of Reactive Oxygen Species (ROS)

To determine the ability of the analyzed GCE, KTE and EAE extracts to generate the intracellular production of reactive oxygen species in HaCaT and BJ cells, a fluorogenic H_2_DCFDA dye (Sigma Aldrich, Saint Louis, MO, USA) was used. After passive diffusion of this compound into the cells, it is deacetylated by intracellular esterases to a non-fluorescent compound. In the presence of reactive oxygen species, it is oxidized and transformed into highly fluorescent 2’,7’-dichlorofluorescein (DCF). The procedure described by Nizioł-Łukaszewska et al. was used [80]. To determine the intracellular level of ROS in HaCaT and BJ, cells were seeded in 96 well plates at a density of 1 × 10^4^ cells per each well. Then, cells were cultured in an incubator for 24 h. DMEM medium was removed and replaced with 10 μM H_2_DCFDA dissolved in serum free DMEM medium. HaCaT and BJ cells were incubated in H_2_DCFDA for 45 min and then incubated with GCE, KTE and EAE extracts at the concentration of 10, 100, 250, 500 and 1000 μg/mL. Cells treated with 1 mM hydrogen peroxide (H_2_O_2_) were used as positive controls. DCF fluorescence was measured after 0, 30 and 60 min using a FilterMax F5 microplate reader (Thermo Fisher Scientific, Waltham, MA, USA)) at a maximum excitation of 485 nm and emission spectra of 530 nm.

#### 3.3.4. Determination of Superoxide Dismutase (SOD) Activity

As part of the research, the Colorimetric Superoxide Dismutase Activity Assay Kit (Abcam, ab65354) was also used to determine the effect of extracts from three plants tested in this paper on the activity of the antioxidant enzyme involved in the defense system against reactive oxygen species—superoxide dismutase. The extracts from these plants were analyzed at concentrations of 100 µg/mL and 500 µg/mL. Recombinant human superoxide dismutase 1 protein (Abcam, ab112193) was used to prepare the standard curve. Analyses were performed on 96-well plates (transparent bottom) according to the manufacturer’s instructions. Initially, 200 µL of WST working solution was added to each well. Then, the test samples were prepared by adding 20 µL of enzyme working solution and 20 µL of *E. angustifolium* L., *C. asiatica* L. and *C. ternatea* L. extracts (at final concentrations of 100 and 500 µg/mL). Three different blank samples were also prepared as recommended. First blank sample was prepared by adding 20 µL enzyme working solution and 20 µL H_2_O to the wells. The second blank was prepared using 20 µL of the dilution buffer and 20 µL of the analyzed plant extracts. To the third blank sample, 20 µL of dilution buffer and 20 µL of H_2_O were added. All samples were mixed, shaken and then incubated at 37 °C for 20 min. The absorbance of the samples was then measured using a FilterMax F5 microplate reader (Thermo Fisher Scientific, Waltham, MA, USA) at λ = 450 nm. All samples were prepared in duplicate according to the manufacturer’s instructions. The ability to inhibit SOD activity by the analyzed samples was calculated from the equation:

$$\%\;SOD\;Activity=\frac{\left(Abs\;blank1\;-\;Abs\;blank3\right)\;-\;\left(Abs\;sample\;-\;Abs\;blank2\right)}{\left(Abs\;blank1\;-\;Abs\;blank3\right)}\times \;100$$

### 3.4. Cytotoxicity Analyses

#### 3.4.1. Cell Culture

As part of this work, two cell lines located in different layers of the skin were used. Normal human keratinocytes (HaCaT) cells were obtained from CLS Cell Lines Service (CLS Cell Lines Service GmbH, Eppelheim, Germany) and fibroblasts (BJ) cells (ATCC^®^CRL-2522 ™, ATCC, Manassas, VA, USA) were purchased from the American Type Culture Collection (Manassas, VA 20108, MA, USA). Cell cultures were performed according to the procedure described by Nizioł-Łukaszewska et al. [80,81]. Both cell lines were grown in Dulbecco’s Modification of Eagle’s Medium (DMEM, Biological Industries) with sodium pyruvate, L-glutamine and high glucose content (4.5 g/L). The medium was supplemented with 10% Fetal Bovine Serum (FBS, Gibco, Waltham, MA, USA) and 1% antibiotics (100 U/mL penicillin and 1000 µg/mL streptomycin, Gibco). Cells were maintained in an incubator at 37 °C in a humid atmosphere of 95% air and 5% carbon dioxide (CO_2_).

#### 3.4.2. Cell Viability Assay

To perform cytotoxicity tests on the tested cell lines, methods described by Nizioł-Łukaszewska et al. [80,81] were used. The medium was aspirated and the bottom-attached cells were washed twice with sterile PBS (phosphate buffered saline, Gibco). Then, the cell layer was trypsinized using trypsin/tetraacetylethylenediamine (EDTA) (Gibco) and the detached cells were centrifuged and resuspended in fresh DMEM medium with the aforementioned supplements. In the next step, the cells were plated in 96-well plates. After the cells were attached to the bottom, they were incubated with various concentrations of analyzed extracts at the concentration range of 10–1000 µg/mL. Then, the cells were placed into the incubator and leftfor 24 h. HaCaT and BJ cells cultured in DMEM without the addition of extracts were used as a control.

#### 3.4.3. Neutral Red Uptake Assay

To evaluate the viability of skin cells treated with GCE, KTE and EAE extracts, the neutral red uptake test (Sigma Aldrich) was used. Tests were carried out in accordance with the procedure previously described by us [80,81]. The average optical density of the control cells (not incubated with analyzed samples) was set to 100% viability and was used to calculate the percentage of viable cells in the experimental samples. The experiments were repeated three times using four wells for each analyzed concentration.

#### 3.4.4. Alamar Blue Assay

To assess the cytotoxicity of GCE, KTE and EAE extracts and evaluate their effect on cell viability, the Alamar Blue (AB) test (Sigma, R7017, Life Tecnologies, Bleiswijk, Netherlands) was used. Test were performed in accordance with the procedure previously described by Nizioł-Łukaszewska et al. [80,81]. After 24 h exposure of HaCaT and BJ cells to analyzed extracts in the concentration range of 10–1000 μg/mL, a solution of resazurin (60 μM) was added to each well. The plates were then incubated at 37 °C for 2 h. After incubation, fluorescence measurements were carried out at λ = 570 nm using a FilterMax F5microplate reader (Thermo Fisher Scientific, Waltham, MA, USA). Three independent experiments were performed to assess cytotoxicity using the AB assay. Results are expressed in graphs as a percentage of cell viability compared to controls (100%).

#### 3.4.5. Lactate Dehydrogenase (LDH) Cytotoxicity Assay

The cytotoxicity of GCE, KTE and EAE extracts was also assessed using the LDH Cytoscan ™ Cytotoxicity Test kit from G-Biosciences (St. Louis, MO, USA). The principle of this assay was based on the conversion of lactate to pyruvate in the presence of lactate dehydrogenase. The test was carried out according to the manufacturer’s instructions and procedure described by Nizioł-Łukaszewska et al. [81]. Analysis was performed on 96-well plates with seeded HaCaT and BJ cells in DMEM medium. After attachment to the bottom, the cells were treated with GCE, KTE and EAE extracts (concentration range of 10–1000 μg/mL). To prepare Spontaneous LDH Activity Control of LDH activity, no compound was added to the wells. After incubation with tested samples, the medium was removed and then the culture supernatant was collected and incubated with 50 μL of reaction mixture for 30 min at 25°C. The reaction was then stopped by adding 50 μL Stop Solution. To determine lactate dehydrogenase activity, absorbance at λ = 490 nm and λ = 680 nm was measured using a FilterMax F5 microplate reader (Thermo Fisher Scientific). Cytotoxicity of the analyzed samples was calculated using the equation:

$$\%\;Cytotoxicity=\frac{Compound\;Treated-Spontaneous\;LDH\;Activity\;}{Maximum\;LDH\;release-Spontaneous\;LDH\;Activity\;}\times 100$$

### 3.5. Determination of Anti-Collagenase Activity

To evaluate the potential anti-aging properties of GCE, KTE and EAE extracts, experiments were carried out to assess the ability of the obtained extracts to inhibit the activity of one of the matrix metalloproteinases—collagenase. For this purpose, the Collagenase Inhibitor Screening Kit (Abcam, ab211108) was used. The water extracts of *Centella asiatica* L., *Clitoria ternatea* L. and *Epilobium angustifolium* L. at the concentrations of 100 and 500 µg/mL were used for the analysis. According to the manufacturer’s instructions and with the procedure described previously by Nizioł-Łukaszewska et al. [81], analyses were performed in a standard 96-well plate with a clear flat bottom. Initially, collagenase (COL) was dissolved in collagenase assay buffer (CAB). The test samples were prepared by mixing GCE, KTE and EAE extracts (separately) at two concentrations with COL and CAB. Inhibitor controls were prepared by mixing inhibitor (1,10-phenanthroline (80 mM) with diluted collagenase and CAB buffer. Enzyme controls were prepared by mixing diluted COL with CAB. The CAB buffer was used as background control. The samples were then incubated for 15 min at room temperature. Meanwhile, a reaction mixture was prepared by mixing the collagenase substrate with CAB. The reaction mixture was then added to all prepared samples and mixed thoroughly. Subsequently, the fluorescence was immediately measured at excitation wavelength λ = 490 nm and emission λ = 520 nm using a microplate reader (FilterMax F5, Thermo Fisher). The measurement was performed in the kinetic mode for 60 min at 37 °C. All samples were prepared in duplicate according to the manufacturer’s instructions. The ability to inhibit COL activity by the analyzed samples was calculated from equation:

$$\%\;relative\;COL\;inhibition=\frac{enzyme\;control-sample}{enzyme\;control}\;\times \;100$$

### 3.6. Determination of Anti-Inflammatory Properties

#### 3.6.1. Inhibition of Protein Denaturation

The ability of obtained extracts to inhibit bovine albumin serum (BSA) denaturation was determined using method described by Sarvesvaran et al. [69]. Then, 1000 µL of plant extracts (100, 500 and 1000 µg/mL) was mixed with 450 µL of 5% aqueous solution of BSA and 1400 µL phosphate buffered saline (PBS). This solution was incubated at 37 °C for 15 min and then heated to 70 °C for 5 min. After heating reaction solutions were cooling in an ice bath to 25 °C and absorbance was measured at 660 nm using the AquamateHelion spectrophotometer (Thermo Fisher Scientific, Waltham, MA, USA). Acetyl salicylic acid (Aspirin, Sigma Aldrich, Saint Louis, MO, USA) was used as positive control (100, 500 and 1000 µg/mL). The percent of inhibition of protein denaturation was calculated from equation:

$$\%\;inhibition\;of\;denaturation=\frac{1-As}{Ac}\;\times \;100$$

where:

*As* is the absorbance of the tested sample,
*Ac* is the absorbance of negative control.
The final result was the arithmetic mean of threeindependent measurements.

#### 3.6.2. Inhibition of Lipoxygenase Activity

The ability of obtained extracts to inhibit lipoxygenase activity was determined using method described by Sarvesvaran et al. [69]. Then, 10 µL of obtained plant extracts (100, 500 and 1000 µg/mL) were mixed in 96-well plate with 160 µL of 100 mM PBS and 20 μL of soybean lipoxygenase solution (167 U/mL). Solutions were incubated at 25 °C for 10 min and then 10 µL of sodium linoleic acid was added to initiate the reaction. The absorbance was measured at 234 nm over a period of 3 min in every minute using a FilterMax F5microplate reader (Thermo Fisher Scientific, Waltham, MA, USA). Quercetin (100, 500 and 1000 µg/mL) was used as positive control. The percent of lipoxygenase activity inhibition was calculated from equation:

$$\%\;inhibition\;of\;lipoxygenase\;activity=\frac{Ac-As}{Ac}\;\times \;100$$

where:

*As* is the absorbance of the tested sample,
*Ac* is the absorbance of negative control.
The final result was the arithmetic mean of fiveindependent measurements.

### 3.7. Transepidermal Water Loss (TEWL) and Skin Hydration Measurements

TEWL and skin hydration measurements were conducted using TEWAmeter TM 300 probe and Corneometer CM 825 probe connected to a MPA adapter (Courage + Khazaka Electronic, Köln, Germany). The study was conducted on 15 volunteers, according to the procedure described by Nizioł-Łukaszewska et al. [81]. Four areas (2 × 2 cm in size) were marked on the forearm skin of volunteers. Then, 0.2 mL of 100 µg/mL solution of obtained extracts was applied to 3 fields. One field (control field) was not treated with any sample. Sample solutions were gently spread over every skin fragment in the marked area and after 20 min dried with a paper towel. After 60, 180 and 360 min, the hydration and TEWL measurements were taken. The final result was the arithmetic mean (from each volunteer) of 5 independent measurements (skin hydration) and 20 measurements (TEWL).

### 3.8. Statistical Analysis

Values of different parameters were expressed as the mean ± standard deviation (SD). The two-way analysis of variance (ANOVA) and Bonferroni post-test between groups were performed at the *p*-value level of <0.05 to evaluate the significance of differences between values. Statistical analysis was performed using GraphPad Prism 8.4.3 (GraphPad Software, Inc., San Diego, CA, USA).

## 4. Conclusions

The analyses of three extracts from *Centella asiatica* L., *Clitoria ternatea* L. and *Epilobium angustifolium* L. carried out as part of this work indicate that the analyzed plants are a valuable source of natural compounds with a broad spectrum of activity. Due to the fact that compounds of natural origin are currently sought, which could replace synthetic substances in preparations for the care and treatment of various skin diseases, such research is needed. The plants analyzed in this paper proved to have strong antioxidant and anti-inflammatory properties, which in the context of the healthy appearance of the skin and preventing various imperfections of the skin is extremely important. In addition, the high demand in the cosmetology and pharmaceutical market for products that have a positive effect on maintaining a youthful appearance and proper hydration of the skin contributed to our evaluation of the obtained extracts in terms of anti-aging activity. The results presented in this study indicate that these plants, especially *Epilobium angustifolium* L., exhibit extremely valuable properties, including antioxidant, anti-inflammatory and anti-aging properties.

## Figures and Tables

**Table 1:** Polyphenols detected using HPLC-ESI-MS in *Epilobium angustifolium* L. (EAE), *Centella asiatica* L. (GCE) and *Clitoria ternatea* L. (KTE) extracts.

No.	Retention Time (min)	Molecular Formula	Molar Mass (Da)	Precursor Ion *m*/*z*	Main Product Ions MS^2^ *m*/*z*	Identification	EAE	GCE	KTE
1	1.6	C_7_H_12_O_6_	192.2	191 [M-H]^−^	127 [M-H-H_2_O-HCOOH]^−^, 85 [M-C_3_H_7_O_4_]^−^, 59 [M-C_5_H_9_O_4_]^−^	Quinic acid	x	x	x
2	2.1	C_7_H_6_O_5_	170.1	169 [M-H]^−^	151 [M-H-H_2_O]^−^, 125 [M-H-CO_2_]^−^, 107 [M-H-CO_2_-H_2_O]^−^, 83 [M-C_3_H_3_O_3_]^−^	Gallic acid	x	-	-
3	2.8	C_16_H_18_O_9_	354.3	353 [M-H]^−^	191 [M-3H_2_O-C_6_H_5_O_2_]^−^, 179 [M-3H_2_O-C_6_H_4_-COOH]^−^	5-Caffeoylquinic acid	x	x	-
4	4.2	C_16_H_18_O_9_	354.3	353 [M-H]^−^	191 [M-3H_2_O-C_6_H_5_O_2_]^−^, 179 [M-3H_2_O-C_6_H_4_-COOH]^−^	3-Caffeoylquinic acid	x	x	-
5	5.1	C_9_H_8_O_4_	180.2	179 [M-H]^−^	135 [M-COOH]^−^, 107 [M-C_3_H_5_O_2_]^−^, 71 [M-C_6_H_5_O_2_]^−^, 59 [M-C_7_H_5_O_2_]^−^	Caffeic acid	x	x	x
6	6.8	C_27_H_30_O_16_	610.5	609 [M-H]^−^	300 [M-H-C_12_H_21_O_9_]^−^,	Rutin	-	-	x
7	7.5	C_27_H_30_O_15_	594.5	593 [M-H]^−^	383 [M-C_8_H_19_O_6_]^−^, 352 [M-C_9_H_21_O_7_]^−^, 284 [M-C_12_H_22_O_9_]^−^	Kaempferol-3-O-rutinoside	-	-	x
8	7.5	C_25_H_24_O_12_	516.4	515 [M-H]^−^	353 [M-C_9_H_7_O_3_]^−^, 335 [M-C_9_H_9_O_4_]^−^, 179 [M-C_16_H_17_O_8_]^−^	3,4-Dicaffeoylquinic acid	x	-	-
9	8.0	C_15_H_10_O_7_	302.2	301 [M-H]^−^	272 [M-CHO]^−^, 255 [M-H-CO-H_2_O]^−^, 151 [M-C_8_H_7_O_3_]^−^, 121 [M-C_8_H_5_O_5_]^−^, 107 [M-C_9_H_5_O_5_]^−^	Quercetin	x	x	x
10	9.9	C_23_H_22_O_13_	506.4	505 [M-H]^−^	300 [M-H_2_O-C_6_H_8_O_4_-COCH_3_]^−^, 271 [M-H_2_O-C_6_H_9_O_4_-COCH_3_-CHO]^−^, 255 [M-C_6_H_15_O_8_], 179 [M-C_15_H_7_O_6_-COCH_3_]	Quercetin-acetyl-glucoside	x	x	x
11	11.3	C_21_H_20_O_11_	448.3	447 [M-H]^−^	284 [M-H_2_O-C_6_H_10_O_4_]^−^, 255 [M-C_6_H_9_O_7_]^−^, 179 [M-C_15_H_9_O_5_]^−^	Kaempferol-3-O-glucoside	x	x	x

**Table 2:** Quantification results obtained for *Epilobium angustifolium* L. (EAE), *Centella asiatica* L. (GCE) and *Clitoria ternatea* L. (KTE) extracts.Values are means±SD of triplicate.

Compound	Content [µg/mL]		
	EAE	GCE	KTE
Quinic acid	220.7 ± 11.7	218.0 ± 12.5	43.1 ± 3.1
Gallic acid	93.7 ± 14.0	<LOD	<LOD
Caffeic acid	0.4 ± 0.0	0.3 ± 0.0	0.3 ± 0.0
5-CQA	55.1 ± 6.0	26.3 ± 1.4	1.2 ± 0.0
3-CQA	47.7 ± 6.7	10.9 ± 0.4	3.4 ± 0.6
Quercetin	50.0 ± 1.4	1.1 ± 0.0	0.6 ± 0.0
Sum of Quantified Compounds	467.6	256.6	75.6

**Table 3:** Total phenolic and flavonoid content of *Centella asiatica* L., *Clitoria ternatea* L., *Epilobium angustifolium* L. of water-ethanol extracts. Values are the mean of three replicate determinations (n = 3) ± SD.

Chemical Compound	TPC [mg GAE/g DW]	TFC [mg QE/g DW]
*Centella asiatica* L.	2.96 ± 0.08	0.82 ± 0.04
*Clitoria ternatea* L.	15.62 ± 0.14	7.26 ± 0.12
*Epilobium angustifolium* L.	12.03 ± 0.16	3.21 ± 0.16

## Data Availability

Data is contained within the article.

## Sample Availability

Samples of the compounds used and the plant extracts tested are available from the authors.

## Appendix A

**Table A1:** Optimized parameters for the LC-ESI-MS/MS quantitative analysis of phenolic acids and flavonoids.

Compund	Precursor Ion *m*/*z* [M-H]^−^	Product Ion, *m*/*z* [M-H]^−^	DP, V	EP, V	CE, V	CXP, V
Quinic acid	190.96	92.9 *	−70	−10	−32	−5
		126.8	−70	−10	−26	−7
Gallic acid	168.919	124.7 *	−60	−10	−22	−5
		78.8	−60	−10	−32	−11
5-Caffeoylquinic acid	353.023	190.8 *	−60	−10	−24	−9
		84.7	−60	−10	−66	−11
3-Caffeoylquinic acid	353.023	190.8 *	−70	−10	−26	−13
		178.9	−70	−10	−28	−13
Caffeic acid	178.952	134.7 *	−60	−10	−24	−7
		134.2	−60	−10	−36	−1
Quercetin	300.903	150.8 *	−115	−10	−30	−7
		178.6	−115	−10	−26	−7

* Transition used for quantification.
